# Supplementary material for: Infants recruit logic to learn about the social world
Source: Nat Commun. 2020 Nov 26;11:5999. doi: 10.1038/s41467-020-19734-5 (PMC7691498; doi:10.1038/s41467-020-19734-5)
Supplement: Supplementary file 1 — Supplementary Information [file 41467_2020_19734_MOESM1_ESM.pdf]

Supplementary Information For

INFANTS RECRUIT LOGIC TO LEARN ABOUT THE SOCIAL WORLD

Nicolò Cesana-Arlotti*, Ágnes Melinda Kovács, Ernő Téglás

* Correspondence to: nicolocesanaarlotti@gmail.com

This PDF file includes:

Supplementary Note 1 to 4

Supplementary References

SUPPLEMENTARY NOTE 1

In order to check for the potential effects of the type of the familiar goal-object, for each experiment we run a preliminary two-way repeated measures ANOVA with type of the choice (consistent choice/inconsistent choice) as a within-participants factor and object type (Ball/Car) as a between-participants factor. In Experiment 1, the analysis detected a main effect of type of the choice ($F(1, 22) = 6.4$, $P = 0.018$) and no main effect of the object type ($F(1, 22) = 0.27$, $P = 0.6$) and no interaction ($F(1, 22) = 0.87$, $P = 0.35$). In Experiment 2, the analysis detected a main effect of type of choice ($F(1, 22) = 28.9$, $P = 0.0001$) and no main effect of the object type ($F(1, 22) = 0.1$, $P = 0.75$) and no interaction ($F(1, 22) = 0.02$, $P = 0.88$). In Experiment 3, the analysis detected a main effect of type of choice ($F(1, 22) = 7.6$, $P = 0.011$) and no main effect of the object type ($F(1, 22) = 0.32$, $P = 0.57$) and no interaction ($F(1, 22) = 0.28$, $P = 0.6$). In Experiment 4, the analysis detected a main effect of type of choice ($F(1, 22) = 7.5$, $P = 0.011$) and no main effect of the object type ($F(1, 22) = 1.29$, $P = 0.26$) and no interaction ($F(1, 22) = 0.83$, $P = 0.37$).

We ran ANOVAS to compare infants' responses to the test conditions between experiments. First, we compared Experiment 1 and 2, to test whether infants looking pattern in Experiment 1 depended on the familiarization with a choice between two objects. A two-way repeated measures ANOVA with type of choice as a within-participants factor and experiment (Experiment1/ Experiment2) as a between-participants factor revealed a main effect of choice type ($F(1, 46) = 8.5$, $P = 0.005$) and a main effect of experiment ($F(1, 46) = 4.1$, $P = 0.047$). Crucially, there was also an interaction between choice type and experiment ($F(1, 46) = 35.3$, $P = 0.0001$), due to the fact that infants in Experiment 1 looked longer at the inconsistent choice (Scheffè test, $P = 0.038$) compared to the consistent one, while those in Experiment 2 looked longer at the consistent choice (Scheffè test, $P = 0.0001$). Second, we compared Experiment 1 and 3, to ask whether infants' responses at the test choices were different when the familiarization competitor was present in the test (Experiment 1) and when it was replaced with a new object (Experiment 3). A two-way repeated measures ANOVA with type of choice as a within-participants factor and experiment (Experiment1/ Experiment3) as a between-participants factor revealed a main effect of choice type ($F(1, 46) = 14.5$, $P = 0.0004$), no main effect of experiment ($F(1, 46) = 2.3$, $P = 0.13$) and no interaction ($F(1, 46) = 0.5$, $P = 0.45$), suggesting that infants' responses to the test conditions in Experiment 1 and 3 were not different. Finally,

we compared Experiment 3 and 4, to ask whether infants' responses at the test choices were different when they were familiarized with a directly visible choice (Experiment 3) and when they had to infer the goal by elimination (Experiment 4). A two-way repeated measures ANOVA with type of choice as a within-participants factor and experiment (Experiment3/ Experiment4) as a between-participants factor revealed a main effect of choice type ($F(1, 46) = 15.8$, $P = 0.0002$), a not significant main effect of experiment ($F(1, 46) = 3.1$, $P = 0.08$) and crucially no interaction ($F(1, 46) = 0.001$, $P = 0.98$), suggesting that the test conditions of Experiment 3 and 4 captured similar looking time patterns.

To check infants' attention during the familiarization of each experiment we measured the total *viewing time* (the time spent looking at the stimuli) during each familiarization trial for each of the four experiments. Given the variation across experiments of the familiarization trial length, we have calculated the proportion of time that the stimuli were attended by a participant (total viewing time divided by trial length). Crucially, in each experiment infants were highly attentive to the familiarization stimuli (mean proportions: $M_{Experiment1} = .97$, $M_{Experiment2} = .96$, $M_{Experiment3} = .97$, $M_{Experiment4} = .93$). To compare infants' attention during familiarization between experiments, we run one-way repeated measures ANOVA with experiment (Experiment1/ Experiment2/ Experiment3/ Experiment4) as a between-participants factor comparing the average proportion of time that infants have spent viewing the familiarization movies. One participant from Experiment 3 was not included in this analysis because of incomplete data due to a technical error. The analysis yielded a main effect of experiment ($F(3, 91) = 4.46$, $P = 0.005$). Infants' average viewing proportion was lower in Experiment 4 than in Experiment 1 (Scheffè test, $P = 0.016$) and then in Experiment 3 (Scheffè test, $P = 0.035$), with no other differences. The slightly lower viewing proportion in Experiment 4 is likely due to the fact that familiarization trials in Experiment 4 were longer compared to the familiarization trials in Experiment 1 and 3. In any case, infants demonstrated the same level of interest toward the familiarization stimuli of Experiment 1 and 2, suggesting that the interaction observed in the response to the test movies of these experiments cannot be explained with a difference in infants' attention to the familiarization stimuli.

In addition, we run Wilcoxon signed-ranks tests to examine individual infants' average looking times in the consistent and inconsistent choice conditions. This analysis converges with the main analysis; in Experiments 1, 3 and 4 infants looked longer at the inconsistent choice than

at the consistent one (Experiment 1: $Z = 1.8$, $P = 0.067$; Experiment 3: $Z = 2.4$, $P = 0.015$; Experiment 4: $Z = 2.8$, $P = 0.005$, all tests were two-tailed), while in the control Experiment 2 they looked longer at the consistent choice than at the inconsistent one (Experiment 2: $Z = -3.9$, $P = 0.0001$, two-tailed). The non-parametric analysis in Experiment 1 likely reflects individual variability that is not uncommon in infant research, and a reduced sensitivity of these tests at our sample size. Importantly, our conclusions are based on the data pattern obtained in four experiments, which consist of one control study (Experiment 2) and two successful conceptual replications of the pattern observed in Experiment 1 (Experiments 3 and 4). Thus, we believe that the initial finding, together with the two conceptual replications, provide a strong support for the proposal we are making aiming to enrich our understanding of infants' logical abilities.

SUPPLEMENTARY NOTE 2

All infants were full term and were recruited on the basis of local birth records. They received a small toy gift for their participation at the end of the experiment. Participants were excluded from the analyses if they contributed with data to only one of the experimental test conditions (inconsistent choice/ consistent choice) or if they had a cumulative looking time of 30 s in more than half of the test trials. A test trial was considered invalid, and not included in the analyses in the following cases: if the caretaker interacted (verbally or otherwise) with the infant and thus not complying with the instructions; the experimenter erroneously triggered the end of a test trial before a 2 s look-away period (as estimated by the offline coding of infants looking behavior); the participant looked at the test outcome for less than 2 cumulative s; or looking time exceeded 2.5 absolute deviations around the median, computed per condition [1].

Based on these criteria, in Experiment 1, an additional eleven infants were tested but not included due to crying or fussiness (3), caretakers' interaction (2), equipment failure (1), experimenter error (1) or insufficient valid samples (4). In Experiment 2, an additional six infants were tested but not retained due to crying or fussiness (1), caretakers' interaction (1), experimenter error (1) or insufficient valid samples (3). In Experiment 3, an additional seven infants were tested but not included due to crying or fussiness (2), caretakers' interaction (1), equipment failure (1), experimenter error (1) or insufficient valid samples (2). In Experiment 4,

an additional seven infants were tested but not retained due to crying or fussiness (3), equipment failure (1), experimenter error (1) or insufficient valid samples (2).

In Experiment 1, the median filter excluded 9% of the trials from analysis. In Experiment 2, the median filter excluded 9% of the trials. In Experiment 3, the median filter excluded 5% of the trials. In Experiment 4, the median filter excluded 4% of the trials.

SUPPLEMENTARY NOTE 3

Each movie was generated at 30 fps. The familiarization movies of Experiments 1 and 3 were 15.3 s long. In Experiment 2, since one object was removed, the amount of object movement before the occlusion was halved, reducing the overall length of each familiarization movie to 13 s. The familiarization movies of Experiment 4 were 18.3 s long. The total length of each familiarization trial was determined by the length of the familiarization movie and a 4 s time interval for which its final event was kept on the screen. The length of the test movies in Experiment 1 to 3 was 18.3 s. The test movies of Experiment 4 were identical to the familiarization movies of Experiment 1 (15.3 s long). The length of the test trial was infant controlled.

Familiarization movies of Experiment 1 and 3 began with displaying two objects (except for Experiment 2, where just one object was displayed) arranged in the center of the scene, horizontally one near the other. In addition, two grey screens were presented at the bottom of the scene. The objects had different colors and shapes and belonged to different categories but had an identical top part. At the beginning of the movies a female voice called for the attention of the infants by saying “Hi baby, hi!”, in Hungarian. Afterwards, both objects pulsed and then moved in a characteristic way accompanied by sounds (e.g., the ball bounced, the car moved back, and the toy telephone rattled), in succession, each for 2 s. Immediately after, each screen covered one object. After one second of full occlusion, the top half of the objects became uncovered, showing that their top part was identical. Afterwards, the occluders simultaneously moved with the objects, one to the lower-left corner of the scene, the other to the lower-right corner, while the objects remained in partial occlusion. After 1 s, the two objects were simultaneously fully uncovered. As soon as the two objects were fully visible, a human hand entered the scene from

the upper edge and stopped for 1.5 s, while infants heard the female voice saying “Look at this!”, in Hungarian. Finally, the hand reached for and grasped one of the two objects, a movement that lasted about 1.5 s.

Test movies of Experiment 1 to 3 began with a display of two objects arranged in the center of the scene, but, unlike in familiarization, they were horizontally positioned one above the other. As in familiarization, two grey screens were presented at the bottom of the scene. Similar to the familiarization, a female voice called for the attention of the infants by saying “Hi baby, hi!”, in Hungarian. Afterward, both objects pulsed and then moved as in familiarization, before the two gray screens closed on the objects as a single joint screen covering both of them. After one second of full occlusion, the top half of the objects became uncovered, showing their identical top parts. While the two objects were now both partly visible, because of the joint occlusion and their shared visual features, their identity could not be determined. Afterwards, the occluders divided and simultaneously moved each with one object, one near the lower-left corner of the scene, the other near the lower-right corner, while the objects remained in partial occlusion. After 1.5 s, one object exited from behind one occluder and moved to the center of the scene, revealing its identity. Crucially, the revealed item was always the non-goal object. At this point, infants could infer by elimination the identity of the other object. The fully visible object briefly pulsed and emitted a sound and then moved back behind the screen where it was before, in the partially occluded position. While the two objects were only partly visible, a human hand entered the scene from the upper edge and stopped for 1.5 s, while a female voice engaged the infant by saying “Look at this!”, in Hungarian. Finally, the hand reached for and grasped one of the two objects, with a movement that lasted 1.5 s.

In Experiments 1 to 3 there were 4 different familiarization movies. The side of the chosen object (left/right) was administered in two orders (LRRLLR/RLLRRL), counterbalanced across participants. For half of the participants, the agent’s goal object was the toy car, for the other half the ball. In Experiments 1 to 3 there were 16 different test movies. The final side of the goal object (Left_Goal-object/Right_Goal-object) and the side of the chosen object (Left/ Right) were administered in two orders (LLRR/RRLL and LRLR/RLRL) counterbalanced across participants. For half of the participants, the initial position of the objects was with the goal object in the upper position, for the other half with the goal object down. For half of the participant the revealed non-goal object was one object, for the other half the other. In

Experiment 4, there were 8 different familiarization movies. The side of the chosen object (Left/Right) and the initial position of the objects (Goal-object_Up/Goal-object_Down) were administered in two orders (LRRLRL/RLLRLR and UUDDUD/DDUUDU) counterbalanced across participants. For half of the participants, the agent's goal object was the toy car, for the other half the ball. In Experiment 4, there were 4 different test movies. The position of the objects (Ball_left/Car_left) and the side of the chosen object (Left/Right) were administered in two orders (BBCC/CCBB and LRLR/RLRL) counterbalanced across participants. In Experiments 1 to 4, the side of the chosen object in the last familiarization movie was the same as in the first test movie for half of the participants, and the opposite for the remaining participants.

SUPPLEMENTARY NOTE 4

Infant' s looking behavior was coded by two experienced coders offline. The primary coder's data were used for the analysis. Inter-coder agreement was calculated for half of the sample. Inter-observer agreement was high for all the four experiments (Experiment 1: $r(40) = 0.96$, $P < 0.05$; Experiment 2: $r(34) = 0.95$, $P < 0.05$, Experiment 3: $r(35) = 0.93$, $P < 0.05$; Experiment 4: $r(45) = 0.99$, $p < 0.05$).

SUPPLEMENTARY REFERENCES

1. Leys, C., Ley, C., Klein, O., Bernard, P. & Licata, L. Detecting outliers: Do not use standard deviation around the mean, use absolute deviation around the median. *Journal of Experimental Social Psychology* **49**, 764–766 (2013).
