# Supplementary material for: Infants recruit logic to learn about the social world
Source: Nat Commun. 2020 Nov 26;11:5999. doi: 10.1038/s41467-020-19734-5 (PMC7691498; doi:10.1038/s41467-020-19734-5)
Supplement: Supplementary file 3 — Reporting Summary [file 41467_2020_19734_MOESM3_ESM.pdf]

nature research

Corresponding author(s): Nicolò Cesana-Arlotti

Last updated by author(s): 9/24/2020

Reporting Summary

Nature Research wishes to improve the reproducibility of the work that we publish. This form provides structure for consistency and transparency in reporting. For further information on Nature Research policies, see Authors & Referees and the Editorial Policy Checklist.

Statistics

For all statistical analyses, confirm that the following items are present in the figure legend, table legend, main text, or Methods section.

n/a	Confirmed	
☐	☒	The exact sample size (n) for each experimental group/condition, given as a discrete number and unit of measurement
☐	☒	A statement on whether measurements were taken from distinct samples or whether the same sample was measured repeatedly
☐	☒	The statistical test(s) used AND whether they are one- or two-sided<br>*Only common tests should be described solely by name; describe more complex techniques in the Methods section.*
☒	☐	A description of all covariates tested
☒	☐	A description of any assumptions or corrections, such as tests of normality and adjustment for multiple comparisons
☐	☒	A full description of the statistical parameters including central tendency (e.g. means) or other basic estimates (e.g. regression coefficient) AND variation (e.g. standard deviation) or associated estimates of uncertainty (e.g. confidence intervals)
☐	☒	For null hypothesis testing, the test statistic (e.g. F, t, r) with confidence intervals, effect sizes, degrees of freedom and P value noted<br>*Give P values as exact values whenever suitable.*
☒	☐	For Bayesian analysis, information on the choice of priors and Markov chain Monte Carlo settings
☒	☐	For hierarchical and complex designs, identification of the appropriate level for tests and full reporting of outcomes
☐	☒	Estimates of effect sizes (e.g. Cohen's d, Pearson's r), indicating how they were calculated

Our web collection on statistics for biologists contains articles on many of the points above.

Software and code

Policy information about availability of computer code

Data collection	The movies were presented on a 24-inch screen with PsyScope X (http://psy.cns.sissa.it/), which controlled the experiment. To extract exact looking time durations for data analysis looking behavior was coded offline with PsyCode (http://psy.cns.sissa.it/).
Data analysis	Data were analyzed with the DataDesk 8 Statistical Analysis software (https://datadescription.com).

For manuscripts utilizing custom algorithms or software that are central to the research but not yet described in published literature, software must be made available to editors/reviewers. We strongly encourage code deposition in a community repository (e.g. GitHub). See the Nature Research guidelines for submitting code & software for further information.

Data

Policy information about availability of data

All manuscripts must include a data availability statement. This statement should provide the following information, where applicable:

- Accession codes, unique identifiers, or web links for publicly available datasets
- A list of figures that have associated raw data
- A description of any restrictions on data availability

The data that support the findings of this study are available in the OSF repository, http://osf.io/adbfs . The source data underlying Fig. 2 is provided as a Source Data file. A reporting summary for this Article is available as a Supplementary Information file.

Field-specific reporting

Please select the one below that is the best fit for your research. If you are not sure, read the appropriate sections before making your selection.

☐ Life sciences ☒ Behavioural & social sciences ☐ Ecological, evolutionary & environmental sciences

For a reference copy of the document with all sections, see nature.com/documents/nr-reporting-summary-flat.pdf

Behavioural & social sciences study design

All studies must disclose on these points even when the disclosure is negative.

Study description	The study aims to assess whether infants can use a logical inference as a source of evidence to evaluate the actions of an observed agents and to acquire new knowledge about her preferences. The study is a quantitative experimental study.
Research sample	A total of 96 full-term , typically developing 14-month-old infants were included in the analysis: Experiment 1 (N = 24; Mage = 14m 02d, range 13m 15d - 14m 14d; 16 girls), Experiment 2 (N = 24; Mage = 14m 02d, range 13m 16d - 14m 16d; 10 girls), Experiment 3 (N = 24; Mage = 14m 06d, range 13m 19d - 14m 15d; 13 girls), Experiment 4 (N = 24; Mage = 13m 29d, range 13m 14d - 14m 11d; 9 girls). All infants were recruited from the "Közigazgatási és Elektronikus Közszolgáltatások Hivatal" (Population Service Centre), Budapest for research purpose. Participants were recruited on the basis of birth records and no selection criteria was applied except for the ones typically used in previous research (see below non-Participation), thus the final sample is likely to be representative of 14-month-old infants population.
Sampling strategy	The sample size of each experiment was identical and selected prior to the start of data collection based on previous research (Teglas et al. 2016; Cesana-Arlotti et al., 2018; Liu et al., 2017). Infants were selected from the university maintained database based on their age.
Data collection	The experiment took place in a sound-proof room with dimmed lights. Participants were seated on their caregiver's lap, at about 60 cm distance from the display. The caregivers wore opaque glasses that prevented them from seeing the stimuli. They were instructed to keep the child seated on their laps and not to interact with them. The experimenter seated behind a curtain and monitored infants' behavior, from a separate screen via a video camera. The experimenter collecting the data was blinded to the order of presentation of the experimental conditions. The entire session was video-recorded. To extract exact looking time durations for data analysis looking behavior was coded off-line by two experienced coders. The primary coder was blind to the study hypothesis and her data were used for the analysis. Inter-coder agreement was calculated for half of the sample. Inter-observer agreement was high for all the four experiments (Experiment 1: $r(40) = 0.96$, $P < 0.05$; Experiment 2: $r(34) = 0.95$, $P < 0.05$, Experiment 3: $r(35) = 0.93$, $P < 0.05$; Experiment 4: $r(45) = 0.99$, $p < 0.05$). The
Timing	Data collection for Experiment 1 started the 3/6/2017 and stopped the 5/22/2017. Data collection for Experiment 2 started the 9/6/2017 and stopped the 10/19/2017. Data collection for Experiment 3 started the 6/6/2017 and stopped the 8/30/2017. Data collection for Experiment 4 started the 10/24/2017 and stopped the 12/14/2017.
Data exclusions	A trial was considered invalid, and not included in the analyses in the following cases: if the caretaker interacted (verbally or otherwise) with the infant and thus not complying with the instructions; the experimenter erroneously triggered the end of a trial before a 2 s look-away period (as estimated by the off-line coding of infants looking behavior); the participant looked at the outcome for less than 2 cumulative s; or looking time exceeded 2.5 absolute deviations around the median, computed per condition. Exclusion criteria were per-established (Leys et al. 2013).<br>In Experiment 1, the median filter excluded 9% of the trials from analysis. In Experiment 2, the median filter excluded 9% of the trials. In Experiment 3, the median filter excluded 5% of the trials. In Experiment 4, the median filter excluded 4% of the trials.
Non-participation	Participants were excluded from the analyses if they contributed with data to only one of the experimental conditions (inconsistent choice/ consistent choice) or if they had a cumulative looking time of 30 s in more than half of the trials. Based on these criteria, in Experiment 1, an additional eleven infants were tested but not included due to crying or fussiness (3), caretakers' interaction (2), equipment failure (1), experimenter error (1) or insufficient valid samples (4). In Experiment 2, an additional six infants were tested but not retained due to crying or fussiness (1), caretakers' interaction (1), experimenter error (1) or insufficient valid samples (3). In Experiment 3, an additional seven infants were tested but not included due to crying or fussiness (2), caretakers' interaction (1), equipment failure (1), experimenter error (1) or insufficient valid samples (2). In Experiment 4, an additional seven infants were tested but not retained due to crying or fussiness (3), equipment failure (1), experimenter error (1) or insufficient valid samples (2).
Randomization	Participants assignment to the experimental groups was blind to the condition.

Reporting for specific materials, systems and methods

We require information from authors about some types of materials, experimental systems and methods used in many studies. Here, indicate whether each material, system or method listed is relevant to your study. If you are not sure if a list item applies to your research, read the appropriate section before selecting a response.

Materials & experimental systems

n/a	Involved in the study
☒	☐ Antibodies
☒	☐ Eukaryotic cell lines
☒	☐ Palaeontology
☒	☐ Animals and other organisms
☐	☒ Human research participants
☒	☐ Clinical data

Methods

n/a	Involved in the study
☒	☐ ChIP-seq
☒	☐ Flow cytometry
☒	☐ MRI-based neuroimaging

Human research participants

Policy information about studies involving human research participants

Population characteristics	See above
Recruitment	All infants were recruited from the "Közigazgatási és Elektronikus Közszolgáltatások Hivatal" (Population Service Centre), Budapest for research purpose. Based on the Hungarian law 1992. LXVI. the CEU CDC receives the addresses of infants born in specific districts. We send letters to the parents informing them about our research and if interested they can contact us. Our participants were typically developing Caucasian infants from middle to middle-upper class families and it is a question for future research how the effects unfold in different populations.
Ethics oversight	The study was approved by United Ethical Review Committee for Research in Psychology (EPKEB) in Hungary.

Note that full information on the approval of the study protocol must also be provided in the manuscript.
