# Supplementary material for: Infants recruit logic to learn about the social world
Source: Nat Commun. 2020 Nov 26;11:5999. doi: 10.1038/s41467-020-19734-5 (PMC7691498; doi:10.1038/s41467-020-19734-5)
Supplement: Supplementary file 4 — Description of Additional Supplementary Files [file 41467_2020_19734_MOESM4_ESM.pdf]

Description of Additional Supplementary Files

File Name: Supplementary Movie 1
Description: Example of a familiarization movie from Experiment 1. Fully visible choice of the toy car over the ball. The toy car is on the right.

File Name: Supplementary Movie 2
Description: Example of a familiarization movie from Experiment 1. Fully visible choice of the toy car over the ball. The toy car is on the left.

File Name: Supplementary Movie 3
Description: Example of a test movie from Experiment 1. The toy car and the ball are partially hidden. The location of the ball is reveled. The agent chooses the other object. Consistent choice.

File Name: Supplementary Movie 4
Description: Example of a test movie from Experiment 1. The toy car and the ball are partially hidden. The location of the ball is reveled. The agent chooses the ball. Inconsistent choice.

File Name: Supplementary Movie 5
Description: Example of a familiarization movie from Experiment 2. Fully visible grasp of the toy car in isolation.

File Name: Supplementary Movie 6
Description: Example of a familiarization movie from Experiment 3. Fully visible choice of the toy car over the toy telephone.

File Name: Supplementary Movie 7
Description: Example of a familiarization movie from Experiment 4. The toy car and the toy telephone are partially hidden. The location of the toy telephone is reveled. The agent chooses the other object.

File Name: Supplementary Movie 8
Description: Example of a test movie from Experiment 4. Fully visible choice of the ball over the toy car.
